# Association between chromosome 6p21 translocation and asthenozoospermia: A retrospective, observational study

**DOI:** 10.1097/MD.0000000000034318

**Published:** 2023-07-07

**Authors:** Yi Zhang, Peng Zhan, Yanli Wang, Wenjie Tian, Xiao Yang, Xu Wang

**Affiliations:** a Department of Urology, the Second Hospital of Jilin University, Changchun, China.

**Keywords:** asthenozoospermia, chromosomal translocation, genetic counseling, male infertility

## Abstract

Asthenozoospermia (AZS) is the commonest cause of male-related infertility. The patients with AZS easily exhibit infertility, with their wives having spontaneous miscarriages or seeking assisted reproductive treatment. Reciprocal chromosomal translocation (RCT) is an important chromosome structural abnormality and has been reported to affect sperm motility. Genetic counseling for male RCT patients with AZS is still a challenge. This study reported 4 RCT carriers, which were 46,XY,t(1;6) (p36.1;p21), 46,XY,t (6;10) (p21;q11.2), 46,XY,t (6;11) (p21;p15), and 46,XY,t (6;17) (p21;q21), respectively. The association between chromosome 6p21 translocation and AZS is discussed, considering 19 published cases as well. In 6 patients with available semen parameters and 4 patients in this study, all of them were diagnosed with AZS. The *SLC26A8* gene and the *DNAH8* gene located on chromosome 6p21 are closely related to AZS by gene search using OMIM. For the chromosome 6p21 breakpoint, 72 pathogenic genes were found through the DECIPHER search. Gene ontology analysis showed that these target genes have several molecular functions and are strongly involved in various biological processes. The proteins expressed by these genes are involved in multiple cellular components. These results suggest that the breakpoint of chromosome 6p21 in male RCT carriers is closely related to AZS. The breakpoint may disrupt the structure and function of related genes, resulting in reduced sperm motility. Karyotype analysis should be recommended for AZS patients. Chromosomes and breakpoints involved in RCT should be paid attention to in genetic counseling for patients.

## 1. Introduction

Male infertility is a multifactorial pathological illness,^[1]^ which is affected by genetic factors, environmental factors, occupational factors, etc.^[2,3]^ In clinical practice, male infertility is diagnosed as azoospermia, oligozoospermia, asthenozoospermia (AZS), teratozoospermia, or multiple abnormal conditions through conventional semen analysis. The genetic factors related to these pathological conditions include chromosome abnormalities, azoospermia factor microdeletion, genetic variations, epigenetic changes, etc.^[4–6]^ Reciprocal chromosomal translocation (RCT) is an important chromosome structural abnormality and has been reported to affect sperm number, morphology, and motility.^[7,8]^

AZS is the commonest causes of male-related infertility^[9]^ and often diagnosed due to reduced sperm motility.^[10]^ AZS patients account for 18% of infertile men.^[11]^ AZS is often observed with decreased sperm count and/or abnormal sperm morphology, which is called oligoasthenospermia, asthenoteratozoospermia, or oligoasthenoteratozoospermia.^[12]^ The patients with AZS easily exhibit infertility, their wife having spontaneous miscarriages or seeking assisted reproductive treatment. Zhuang et al^[13]^ reported 30 patients with AZS were treated with intracytoplasmic sperm injection and achieved good results. Yovinska et al^[14]^ reported that chromosomal translocations were tightly associated with oligoasthenozoospermia, Hence, genetic counseling for male RCT patients with AZS is still a challenge.

This study reported 4 males with chromosome 6p21 translocation. Moreover, the association between chromosome 6p21 translocation and AZS has been discussed considering published cases as well.

## 2. Materials and methods

### 2.1. Study design and settings

An observational, retrospective study was conducted at the Second Hospital, Jilin University. This study was approved by the Ethics Committee of the Second Hospital, Jilin University. The need for informed consent was waived because of the retrospective design of this study.

### 2.2. Patients

The subjects of this study included 4 male RCT carriers, all of whom were patients who went to the andrology clinic for consultation because their wives had not given birth for many years after marriage. These patients’ medical histories were enquired, and each one had a physical examination to ascertain their height, weight, and testis volume. The pertinent examinations of each patient’s spouse were also recorded at the same time. These patients had not been exposed to teratogenic substances, radiation, or infectious disorders, nor had their partners.

### 2.3. Semen analysis

Semen analysis was performed by 2 professional technicians according to the method recommended by the World Health Organization guidelines.^[15]^ Semen collection and sperm parameter detection are performed by our method described previously.^[16]^ All patients underwent semen analysis more than twice. AZS was diagnosed when the percentage of progressive sperm in semen was lower than the reference value of 32%.

### 2.4. Reproductive hormonal analyses

Reproductive hormones were examined in blood samples from 4 patients. After being kept at room temperature for 30 minutes, blood samples were centrifuged at 1000 × *g* for 10 minutes. Until analysis, serum was kept in sterile tubes at −20 °C. An electrochemiluminescence immunoassay was used to measure the levels of serum follicle-stimulating hormone, luteinizing hormone, and testosterone (Roche Diagnostics, Mannheim, Germany).

### 2.5. Cytogenetic analysis

Peripheral blood collection, culture, and G-banding are carried out according to the method described previously.^[7]^ The karyotypes were described according to the International System for Human Cytogenetic Nomenclature (ISCN 2020).

### 2.6. Literature review and GO analysis

To explore the relationship between male RCT and semen parameters, relevant studies involving chromosome 6p21 translocation were searched in PubMed. Cases with breakpoints on chromosome 6p21 were collected. To evaluate the relationship between translocation breakpoints and clinical phenotype, related genes on chromosomes 1p36, 6p21, 10q11, 11p15, and 17q21 were searched using Online Mendelian Inheritance in Man (OMIM; https://www.ncbi.nlm.nih.gov/omim). For chromosome 6p21 breakpoints that existed in all cases in this study, 72 pathogenic genes were found through DECIPHER search (https://www.deciphergenomics.org/). Then, gene ontology (GO) analysis was performed for these 72 pathogenic genes (https://david.ncifcrf.gov/home.jsp).

## 3. Results

**Figure 1.**
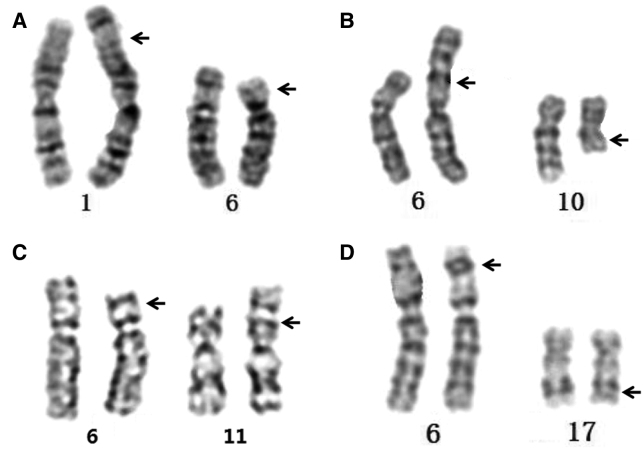
G-banding karyotypes of the 4 patients in this study (A: Case 1; B: Case 2; C: Case 3; D: Case 4).

Four male infertile individuals were involved in this study. Table 1 displays the clinical information of 4 patients. Case 1 involved a 36-year-old man who had a normal phenotypic and was identified as having AZS. In the 2 years following their marriage, his spouse had not given birth. A 32-year-old male in Case 2 was identified as having asthenoteratozoospermia despite having a normal phenotypic. Case 3 had a 28-year-old man who had a normal phenotypic and was identified as having AZS. In Case 4, a 30-year-old male with a normal phenotypic had oligoasthenoteratozoospermia as determined by semen analysis. The cytogenetic analysis yielded the following results: 46,XY,t(1;6)(p36.1;p21), 46,XY,t(6;10)(p21;q11.2), 46,XY,t(6;11)(p21;p15), and 46,XY,t(6;17)(p21;q21), respectively (Fig. 1A–D). Their wives had healthy chromosomes (46,XX). When their spouses underwent routine clinical evaluations, no untoward alterations were found. Four patients’ parents were suggested for a cytogenetics investigation. Only the parents of Case 4 undertook karyotype analysis after giving their informed consent; the results revealed that his mother’s karyotype was 46,XX, t(6;17) (p21; q21) and his father’s chromosome was 46, XY. All couples selected IVF and preimplantation genetic diagnosis as their methods of assisted conception following genetic counseling. While Cases 3 and 4 are continuing in the assisted pregnancy cycle, Cases 1 and 2 have already given birth to offspring with normal phenotypes.

**Table 1:** Clinical information of 4 patients in this study

Variable	Case 1	Case 2	Case 3	Case 4
Age (years)	36	32	28	30
BMI (kg/m^2^)	25.2	22.6	24.5	23.8
Semen volume (mL)	1.6	1.5	1.4	1.6
Sperm concentration (×10^6^/mL)	13	16	15	9
Progressive motility (%)	15	18	12	7
Total motility (%)	26	32	24	19
Sperm morphology (normal form, %)	6	1	12	2
Testicular volume (mL)	Left: 12; Right: 12	Left: 15; Right: 15	Left: 12; Right: 12	Left: 12; Right: 13
FSH (mIU/mL)	3.6	5.7	4.6	2.1
LH (mIU/mL)	1.5	4.3	3.7	5.9
T (nmol/L)	20.3	15.8	17.6	22.4
Clinical finding	Asthenozoospermia	Asthenoteratozoospermia	Asthenozoospermia	Oligoasthenoteratozoospermia
Karyotype	46,XY,t(1;6)(p36.1;p21)	46,XY,t(6;10)(p21;q11.2)	46,XY,t(6;11)(p21;p15)	46,XY,t(6;17)(p21;q21)
Figure	Fig. 1A	Fig. 1B	Fig. 1C	Fig. 1D

BMI = body mass index, FSH = follicle-stimulating hormone, LH = luteinizing hormone, T = testosterone.

A total of 19 cases of chromosomal 6p21 translocation were gathered for this study through a search of the literature. Table 2 displays the seminal characteristics of these carriers and the reproductive history of these spouses. All carriers were AZS, according to the available semen characteristics. These carriers experience a variety of reproductive consequences, including infertility, recurrent abortions, and preimplantation genetic diagnosis treatment.

**Table 2:** Clinical features of RCT carriers involving chromosome 6p21 breakpoints

Case	Karyotype	Seminal parameters	Reproductive history of the couple	Reference
1	t(1;6)(p36.13; p21.31)	N/A	PGD	Ko et al^[17]^
2	t(1;6)(p22; p21.3)	N/A	PGD	Escudero et al^[18]^
3	t(2;6)(p13; p21.3)	N/A	Recurrent abortions	Al-Hussain et al^[19]^
4	t(2;6)(q21; p21)	N/A	Three biochemical pregnancies	Yang et al^[20]^
5	t(2;6)(q37; p21)	N/A	Infertility	Anton et al^[21]^
6	t(4;6)(p16; p21)	NPS-ASP: Poor	N/A	Mayeur et al^[22]^
7	t(4;6)(q21.3; p21)	Asthenozoospermia	N/A	Matsuda et al^[23]^
8	t(5;6)(q35; p21.3)	N/A	PGD	Ko et al^[17]^
9	t(6;8)(p21; q24)	N/A	Spontaneous abortion	Zhang et al^[24]^
10	t(6;8)(p21; q24)	N/A	Two spontaneous abortions	Yang et al^[20]^
11	t(6;10)(p21.1; q24)	NPS-ASP: Very poor	N/A	Mayeur et al^[22]^
12	t(6;10)(p21; q26)	Asthenozoospermia	N/A	Perrin et al^[25]^
13	t(6;10)(p21; q26)	N/A	Repeated miscarriages, PGD	Perrin et al^[26]^
14	t(6;13)(p21.3; q14.3)	N/A	PGD	Zhang et al^[27]^
15	t(6;13)(p21.1; q32)	N/A	Infertility, PGD	Yakut et al^[28]^
16	t(6;15)(p21; q26.1)	N/A	Recurrent fetal wastage	Fryns et al^[29]^
17	t(6;17)(p21; p13)	N/A	Repetitive abortions	Davis et al^[30]^
18	t(6;17)(p21.3; q21)	Oligoasthenoteratozoospermia	N/A	Rouen et al^[31]^
19	t(6;21)(p21.1;pl3)	Oligoasthenoteratospermia	Infertility	Paoloni-Giacobino et al^[32]^

N/A = not applicable, NPS-ASP = the number of progressive spermatozoa retrieved after sperm preparation, PGD = preimplantation genetic diagnosis.

**Figure 2.**
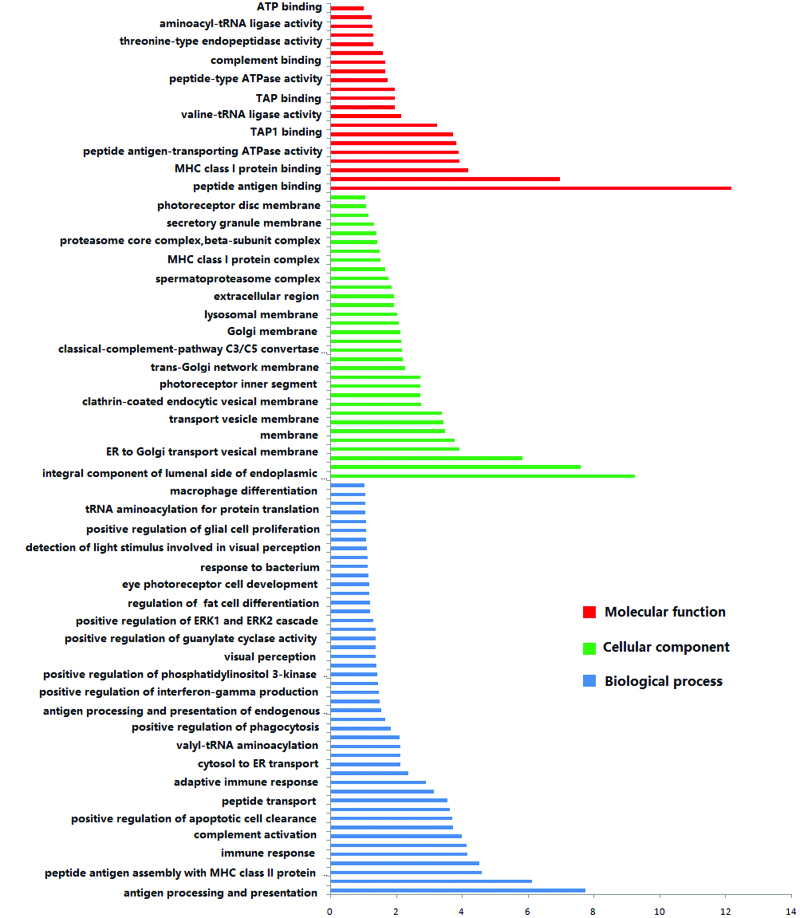
GO analysis results of genes located on chromosome 6p21. Abscissa represents −log (*P* value), and ordinate represents GO terms. GO = gene ontology.

Examining the connection between these breakpoints and clinical phenotype, related genes on chromosome 1p36, 6p21, 10q11, 11p15, and 17q21 were summarized in Table 3. The *SLC26A8* gene and *DNAH8* gene located on chromosome 6p21 are closely related to AZS. GO analysis revealed that these target genes on chromosome 6p21 have multiple molecular functions and are heavily involved in a number of biological processes, which allowed researchers to further investigate the association between these genes and clinical symptoms. Numerous biological components are affected by the proteins that these genes express (Fig. 2). These target genes were implicated in adenosine triphosphate (ATP) binding, among other things, according to the results of molecular functions, which may help to explain the origin of AZS. Various plasma membrane features and extracellular exosomes have an impact on sperm motility.

**Table 3:** Important genes and its functions related to RCT breakpoints in this study

Breakpoint	Gene	Full name of gene	Loci	Expression or function	Clinical findings
1p36	*CATSPER4* (609121)	Cation channel, sperm-associated,4	1p36.11	CATSPER is essential for sperm hyperactivated motility.	N/A
6p21	*SLC26A8* (608480)	Solute carrier family 26, member 8	6p21.31	The physical interactions between SLC26A8 and CFTR are required for sperm motility.	Asthenozoospermia
	*DNAH8* (603337)	Dynein, axonemal, heavy chain 8	6p21.2	DNAH8 is an outer arm axonemal dynein heavy chains.	Asthenoteratozoospermia
10q11	N/A	N/A	N/A	N/A	N/A
11p15	*DNHD1* (617277)	Dynein heavy chain domain 1	11p15.4	DNHD1 is localized along the entire flagella.	Asthenoteratozoospermia
17q21	*KLHL10* (608778)	Kelch- like 10	17q21.2	KLHL10 is expressed exclusively in the cytoplasm of elongating and elongated spermatids	Severe oligozoospermia

N/A = not applicable.

## 4. Discussion

AZS is a clinical manifestation and the most common cause of male infertility.^[33]^ Up to this point, it has been discovered that AZS is linked to environmental factors, energy metabolism, and genetic factors.^[10,34]^ Chromosome abnormalities, particular nuclear and mitochondrial gene mutations, and epigenetic changes are a few examples of genetic causes.^[10,35]^ Although many academics have worked hard, the precise etiology of AZS is still unknown.^[36]^ Sperm motility deficiencies are at risk due to chromosomal translocation.^[8]^ Sperm motility and progressive motility were considerably lower in RCT carriers than in control groups.^[37]^ However, RCT carriers and the control group did not differ in terms of progressive sperm motility and motility grade D, according to Pastuszek et al’s^[38]^ research. Therefore, more research on the connection between chromosomal translocation and AZS is still necessary.

Four male RCT patients were identified in this investigation as having AZS, according to our findings (Table 1). These RCT carriers have a breakpoint on chromosome 6p21. Nineteen documented instances were gathered in order to further investigate the connection between RCT harboring 6p21 and AZS (Table 2). All of the patients in this study’s 4 patients and the 6 patients whose semen data were available exhibited decreased sperm motility. Further research should be done on the exact causes.

Previous studies have shown that the breakpoint of translocation disrupts the structure and function of important genes related to spermatogenesis.^[39]^ Related genes on chromosome breakpoints of this study were summarized in Table 3. The *SLC26A8* gene, which is located at chromosome 6p21.31, is associated with human AZS.^[40]^ The *DNAH8* gene has been mapped to chromosome 6p21.2, and is related to AZS.^[41]^ Hence, we speculated that AZS of this study may have been related to the disruption of the *SLC26A8* or/and *DNAH8* gene. Meanwhile, the result that the GO analysis suggested that these target genes were involved in extracellular exosome, various plasma membrane structures, and ATP binding, etc. Exosomes can improve spermatozoa motility.^[42]^ The transport of small vesicles emanating from the Golgi cisternae to the plasma membrane is related to ATP production, which is responsible for sperm motility.^[43]^ These studies may explain the cause of AZS for RCT carriers. To be sure, more complex reasons may still exist, and further study is expected.

In addition, seminal parameters are not available in some cases, and the wives of RCT carriers have recurrent spontaneous abortions (Table 2). A meta-analysis result showed that current research supports an association between the sperm deoxyribonucleic acid (DNA) fragmentation index and recurrent pregnancy loss.^[44]^ Some studies showed that men with chromosomal structural abnormality had a higher rate of sperm DNA fragmentation.^[45,46]^ Tang et al^[47]^ reported that sperm DNA fragmentation index had predictive value for IVF fertilization of men with mild-to-moderate AZS. More detailed cases should be collected to clarify the relationship between RCT, AZS, sperm DNA damage, and recurrent spontaneous abortion.

The limitations of this study include: molecular genetic testing was not performed. The number of cases involved in chromosome 6p21 translocation is small and sperm parameters of some reported cases are not available.

## 5. Conclusions

In conclusion, we report 4 male RCT carriers involving the breakpoint of 6p21 and review 19 reported cases of the same chromosome band. Among them, the cases with available semen parameters were diagnosed as AZS. The chromosome 6p21 breakpoint may disrupt the structure and function of related genes, resulting in reduced sperm motility. However, the clinical outcomes of these RCT carriers are varied. Karyotype analysis should be recommended for AZS patients. Chromosomes and breakpoints involved in RCT should be paid attention to in genetic counseling for patients.

## Author contributions

**Conceptualization:** Yi Zhang, Peng Zhan.

**Data curation:** Yanli Wang, Wenjie Tian.

**Investigation:** Yi Zhang, Peng Zhan, Wenjie Tian.

**Methodology:** Xiao Yang, Xu Wang.

**Writing – original draft:** Yi Zhang.

**Writing – review & editing:** Peng Zhan.
